# Supplementary material for: Dietary Cholic Acid Exacerbates Liver Fibrosis in NASH Model of Sprague–Dawley Rats Fed a High-Fat and High-Cholesterol Diet
Source: Int J Mol Sci. 2022 Aug 17;23(16):9268. doi: 10.3390/ijms23169268 (PMC9409005; doi:10.3390/ijms23169268)
Supplement: Supplementary file 1 [file ijms-23-09268-s001.zip › ijms-1857475-supplementary.pptx]

## Slide 1
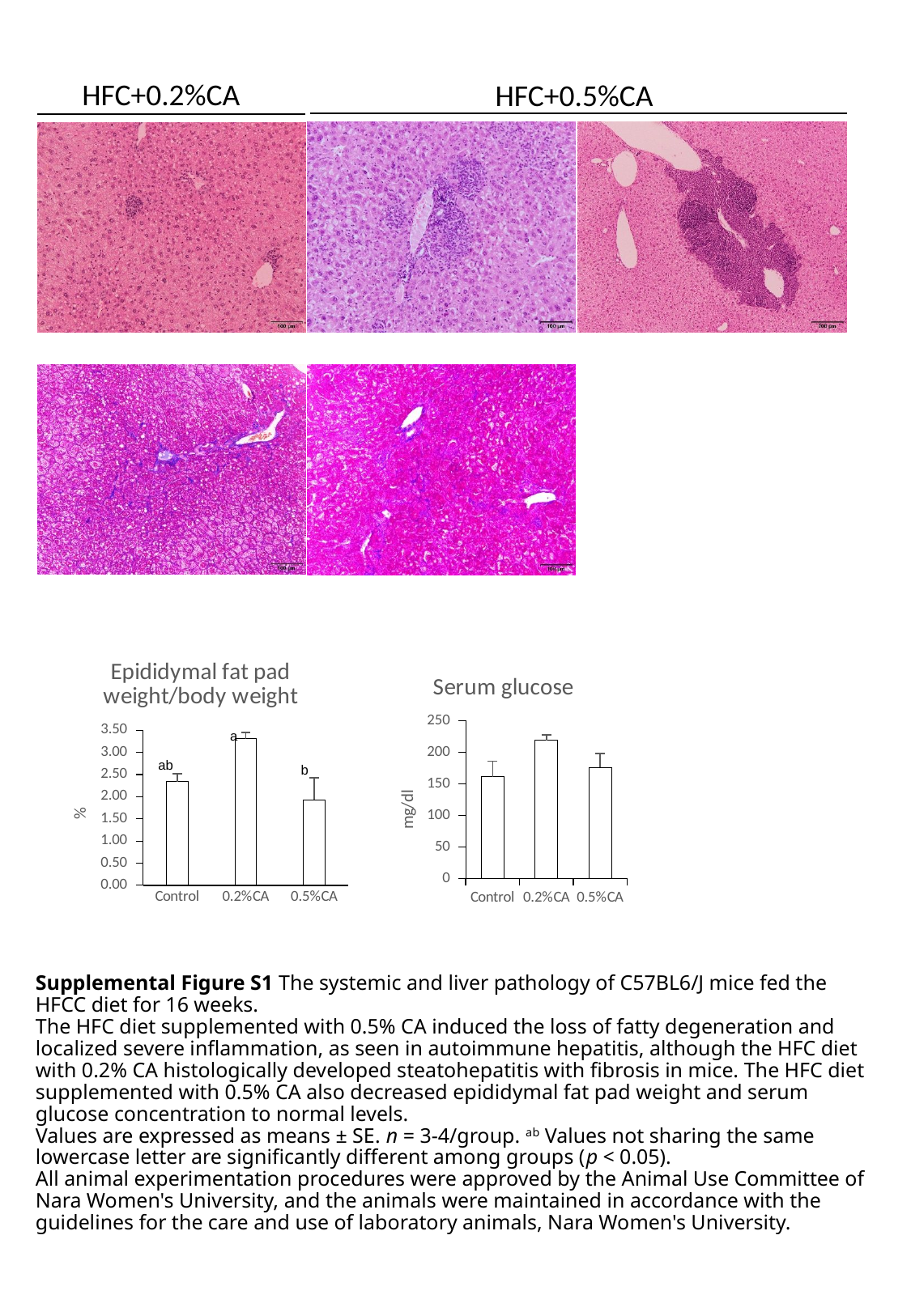

HFC+0.2%CA
HFC+0.5%CA
### Chart: Epididymal fat pad weight/body weight
| Category | |
|---|---|
| Control | 2.34 |
| 0.2%CA | 3.304836098922014 |
| 0.5%CA | 1.9314963684278033 |
### Chart: Serum glucose
| Category | Glu |
|---|---|
| Control | 161.11266009852216 |
| 0.2%CA | 218.8460591133005 |
| 0.5%CA | 175.85550082101804 |a
ab
b
Supplemental Figure S1 The systemic and liver pathology of C57BL6/J mice fed the HFCC diet for 16 weeks.
The HFC diet supplemented with 0.5% CA induced the loss of fatty degeneration and localized severe inflammation, as seen in autoimmune hepatitis, although the HFC diet with 0.2% CA histologically developed steatohepatitis with fibrosis in mice. The HFC diet supplemented with 0.5% CA also decreased epididymal fat pad weight and serum glucose concentration to normal levels.
Values are expressed as means ± SE. n = 3-4/group. ab Values not sharing the same lowercase letter are significantly different among groups (p < 0.05).
All animal experimentation procedures were approved by the Animal Use Committee of Nara Women's University, and the animals were maintained in accordance with the guidelines for the care and use of laboratory animals, Nara Women's University.
